# Vascular Inflammation and Oxidative Stress: Major Triggers for Cardiovascular Disease

**DOI:** 10.1155/2019/7092151

**Published:** 2019-06-23

**Authors:** Sebastian Steven, Katie Frenis, Matthias Oelze, Sanela Kalinovic, Marin Kuntic, Maria Teresa Bayo Jimenez, Ksenija Vujacic-Mirski, Johanna Helmstädter, Swenja Kröller-Schön, Thomas Münzel, Andreas Daiber

**Affiliations:** ^1^Center for Cardiology, Cardiology I-Laboratory of Molecular Cardiology, University Medical Center of the Johannes Gutenberg-University, Mainz, Germany; ^2^Center for Thrombosis and Hemostasis (CTH), University Medical Center of the Johannes Gutenberg-University, Mainz, Germany; ^3^German Center for Cardiovascular Research (DZHK), Partner Site Rhine-Main, Mainz, Germany

## Abstract

Cardiovascular disease is a leading cause of death and reduced quality of life, proven by the latest data of the Global Burden of Disease Study, and is only gaining in prevalence worldwide. Clinical trials have identified chronic inflammatory disorders as cardiovascular risks, and recent research has revealed a contribution by various inflammatory cells to vascular oxidative stress. Atherosclerosis and cardiovascular disease are closely associated with inflammation, probably due to the close interaction of inflammation with oxidative stress. Classical therapies for inflammatory disorders have demonstrated protective effects in various models of cardiovascular disease; especially established drugs with pleiotropic immunomodulatory properties have proven beneficial cardiovascular effects; normalization of oxidative stress seems to be a common feature of these therapies. The close link between inflammation and redox balance was also supported by reports on aggravated inflammatory phenotype in the absence of antioxidant defense proteins (e.g., superoxide dismutases, heme oxygenase-1, and glutathione peroxidases) or overexpression of reactive oxygen species producing enzymes (e.g., NADPH oxidases). The value of immunomodulation for the treatment of cardiovascular disease was recently supported by large-scale clinical trials demonstrating reduced cardiovascular mortality in patients with established atherosclerotic disease when treated by highly specific anti-inflammatory therapies (e.g., using monoclonal antibodies against cytokines). Modern antidiabetic cardiovascular drugs (e.g., SGLT2 inhibitors, DPP-4 inhibitors, and GLP-1 analogs) seem to share these immunomodulatory properties and display potent antioxidant effects, all of which may explain their successful lowering of cardiovascular risk.

## 1. Inflammation and the Global Burden of Disease and Age-Related Cardiometabolic Complications

### 1.1. Cardiovascular Risk Factors and Global Burden of Disease: Contribution of Inflammation

Endothelial (vascular) dysfunction is an early correlate for coronary artery disease in humans [1] and occurs in many low-grade inflammatory diseases, including rheumatoid arthritis [2, 3], psoriasis [4], and type 2 diabetes [5] and thereby accelerates atherosclerosis and causes cardiovascular mortality. Endothelial dysfunction is also found in severe inflammatory conditions, such as lipopolysaccharide- (LPS-) induced septic shock [1]. The PROVE IT-TIMI 22 study demonstrated that high CRP levels in patients with acute coronary syndrome predicted death from myocardial infarction [6], which adds further support to the striking correlation found between inflammation and increased cardiovascular disease risk (reviewed in [7–9]) and also provides strong evidence for a close interaction between inflammation, oxidative stress, and redox signaling, presenting a rich field for future investigation. Inflammation plays also a central role in neurodegenerative processes such as Alzheimer's disease [10–12] and Parkinson's disease [13, 14]. Accordingly, inflammatory disorders represent a major challenge for health care systems and societies worldwide.

**Figure 1:**
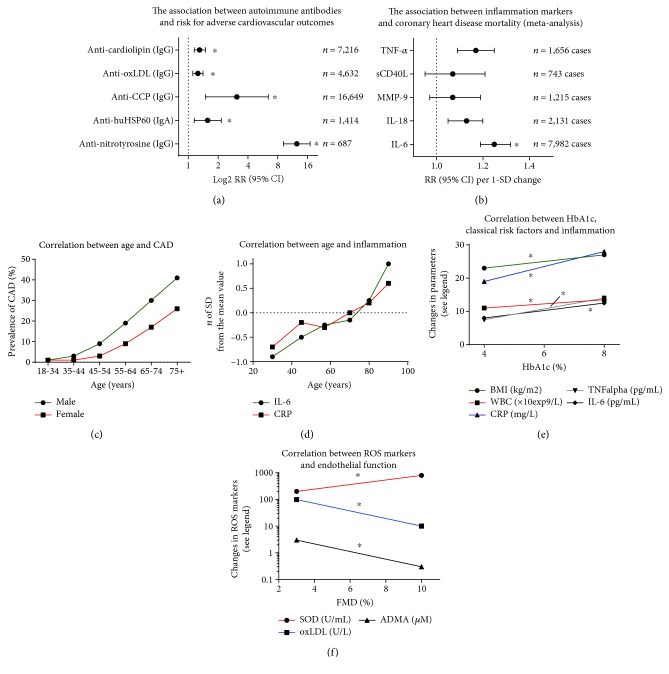
Impact of autoimmune antibodies and inflammation markers on cardiovascular events or mortality—associations between age or glycemic state and inflammation. (a) Hazard ratios for adverse cardiovascular outcomes in correlation with autoimmune antibodies (IgG subtype) obtained by meta-analysis and adjustment for age, sex, smoking status, adiposity markers, blood pressure, and/or lipid markers (number of cases as indicated) and adjustment for classical confounders. ∗ indicates significant differences to the control group. oxLDL = oxidized low-density lipoprotein; CCP = cyclic citrullinated protein; HSP60 = heat shock protein 60. Graph was generated from tabular data by Thomson et al. [284] for anti-nitrotyrosine or Iseme et al. [18] for all other autoimmune antibodies. (b) Hazard ratios for all coronary heart disease mortality in correlation with markers of inflammation interleukin- (IL-) 6, IL-18, matrix metalloproteinase- (MMP-) 9, soluble CD40 ligand (sCD40L or CD154), and tumor necrosis factor- (TNF-) *α* obtained by meta-analysis and adjustment for age, sex, smoking status, adiposity markers, blood pressure, and/or lipid markers (number of cases as indicated). Risk increases are shown per 1 SD changes of cytokines. ∗ indicates significant differences to the control group. Redrawn from tabular data by Kaptoge et al. [19]. (c) Prevalence of coronary artery diseases (CAD) increases with the progressing age and gender in the general German population. Drawn from results of the DETECT study [29]. (d) Mean values of inflammatory markers (CRP = C-reactive protein and IL-6) according to the age group expressed as number of standard deviations from the sex-specific population mean to make them independent of different units of measure. Only data for men (*n* = 595) are shown, but those for women (*n* = 748) look very similar. Other markers of inflammation show similar correlations (e.g., IL-18 and fibrinogen). Graph was roughly estimated from tabular data by Ferrucci et al. [50]. (e) The correlation between HbA1c and potential risk factors. The correlation between HbA1c and body mass index (BMI), white blood cells (WBC), CRP, TNF-*α*, and IL-6 (*n* = 221 subjects). Graph was roughly estimated from graphical data by Wang et al. [62]. (f) The correlation between serum superoxide dismutase (SOD) activity, oxLDL and asymmetric dimethylarginine (ADMA) levels, and endothelial function measured by FMD (*n* = 59 healthy and chronic kidney disease subjects). Graph was roughly estimated from graphical data by Yilmaz et al. [117]. ∗ indicates significant differences to the control group.

The Global Burden of Disease Study (GBD) from 2012 illustrates a significant shift in the factors impacting life expectancy from communicable childhood disease toward noncommunicable diseases common in the aged [15, 16]. This study also found that collectively, hypertension, ischemic heart disease, smoking, and cerebrovascular disease accounted for more than 50% of global deaths. These diseases and risk factors are also the leading causes of premature mortality and responsible for more than 20% of life years lost due to severe disability [15, 16]. According to a more recent update of the GBD, diabetes and related metabolic diseases are experiencing a continuous increase in prevalence as well as incidence. Diabetes (high fasting blood glucose), hyperlipidemia (high total cholesterol), and obesity (high body mass index) rank on positions 3, 4, and 7, respectively, on the list of leading health risk factors [17]. The fact that 4 risk factors related to cerebro/cardiovascular health problems and 3 cardiometabolic risk factors were identified among the leading causes of global mortality underlines the importance of adequate cardiovascular therapy. Although inflammatory processes are not explicitly mentioned in the Global Burden of Disease Study (only lower respiratory infections are listed among the 14 most important risk factors for global deaths [15, 16]), the contribution of dysregulated immune responses and chronic (low-grade) inflammation to atherosclerosis and subsequently cardiovascular disease development and progression is well accepted and documented (see the hazard ratio for markers of inflammation/atherothrombosis such as MMP-9 and sCD40L, autoimmune antibodies, and cardiovascular risk in Figures 1(a) and 1(b)) [18–20]. In the following two sections, two conditions with a low-grade inflammatory phenotype and increasing prevalence will be discussed in detail—aging and diabetes.

### 1.2. Increased Prevalence of Cardiovascular Disease at Higher Age

The number of people older than 65 years is projected to dramatically increase in the next decades, presenting demographic change as an emergent issue in Western society [21]. Demographic shifts toward an older population will have economic effects like increased spending on retirement funds and will also amplify the economic burden for healthcare costs. The expected increase in cardiovascular disease (CVD) burden corresponding to the shift toward an older population creates an urgent need for research in the field to supplement limited existing knowledge in order to reduce the predicted increase in morbidity and mortality. In particular, it is necessary to elucidate the pathophysiological convergence of separate diseases with comorbidities that also increase as age advances [22–24]. CVD is a leading contributor to morbidity and mortality in the elderly and closely correlated with age [25] due to the phenomenon of “vascular aging,” which encompasses all age-associated changes in vessels. Since aged vessels are more prone to atherosclerotic lesions, vascular injury, impaired angiogenesis, and calcification [26], it is clear that the aging endothelium is more and more unable to regulate all its tasks, manifesting as significant impairment of endothelium-dependent relaxation (endothelial dysfunction) in elderly people [27, 28]. This supports the demographic data illustrating a positive correlation between CVD incidence or complications and age, also supported by higher use of CVD medications with increasing age (Figure 1(c)) [29]. Mechanistically, both smooth muscle and endothelial cells are involved in the vascular aging process because aging leads to arterial stiffness and endothelial dysfunction, both known to correlate with future cardiovascular events in humans [30].

Since endothelial dysfunction promotes thrombocyte activation [31], vasoconstriction, leukocyte activation/infiltration [32, 33], and smooth muscle cell proliferation (intima-media thickness) [34] in the vessel wall, it is consequently an early predictor for the development of atherosclerosis, hypertension, and future cardiovascular events (for review, see [9]). Recently, three interdependent key players in the vascular aging process have been identified that can trigger endothelial dysfunction: impaired nitric oxide signaling, oxidative stress, and inflammation [35]. Age-dependent endothelial dysfunction and oxidative stress are found in all vessels, from macrovessels like the aorta and coronary arteries to resistance vessels of the microcirculatory system (for review, see [36]). Paralleling the wide-ranging presence of oxidative stress, endothelial dysfunction in the elderly is not only associated with CVD but also with other disorders related to aging, such as erectile dysfunction, renal dysfunction, Alzheimer's disease, or retinopathy [37–40]. Highlighting this, studies by Mayhan et al. demonstrated an impaired eNOS-dependent reactivity in cerebral arterioles, which was additionally positively associated with increased oxidative stress at higher age [41]. Endothelial dysfunction has been demonstrated in aging retinal vessels [42] and also implicated as a contributor in neurodegenerative disease [43, 44]. Impaired ^·^NO signaling, vascular inflammation, and oxidative stress are key players in the pathogenesis of age-dependent endothelial dysfunction, as demonstrated by our group and many others (for review, see [45, 46]). In combination, these data all implicate aging as an independent risk factor for cardiovascular disease, mainly due to oxidative stress-induced endothelial dysfunction and low-grade inflammation [47, 48].

Plasma concentrations of some inflammatory markers (sVCAM-1, IL-6, MCP-1, and others) are positively correlated with age, independent of other cardiovascular risk factors [49]. In both men and women, older age was associated with higher levels of interleukin-6 (IL-6), IL-1 receptor antagonist (IL-1ra), IL-18, C-reactive protein (CRP), and fibrinogen (most changes persisted after correction for other risk factors), while soluble IL-6 receptor (sIL-6r) increased significantly with age only in men (Figure 1(d)) [50]. A meta-analysis (32 cross-sectional studies, *n* > 23,000 subjects) also revealed that age-associated frailty and prefrailty are associated with higher inflammatory parameters, in particular CRP and IL-6 [51]. Hazard ratios for serum CRP levels as well as IL-6 at baseline and incident of age-associated frailty were 1.06 (95% confidence interval (CI): 0.78-1.44) and 1.19 (95% confidence interval (CI): 0.87-1.62), respectively, after adjustment for 9 potential confounders [51].

### 1.3. Increased Prevalence of Cardiovascular Disease in the Setting of Diabetes and Related Cardiometabolic Disease

A recent estimation by the World Health Organization (WHO) from October 2018 states that “the number of people with diabetes has risen from 108 million in 1980 to 422 million in 2014,” with a more rapid increase in middle- and low-income countries [52]. WHO further states that “in 2016, an estimated 1.6 million deaths worldwide were directly caused by diabetes and another 2.2 million deaths were attributable to high blood glucose in 2012.” This figure is only expected to rise dramatically within the next decades. In addition to being a leading cause of mortality (see recent GBD data [17]), diabetes is the most common cause of blindness, nontraumatic amputations, and end-stage renal disease in adults. The cost associated with this disease is devastating. Compounding the costs of diabetes and heart disease paints a bleaker picture; in China from 2006-2015, a $558 billion loss in national income was attributable to heart disease, stroke, and diabetes. Diabetes mellitus is an accepted independent risk factor in the development of coronary heart disease with a notable adverse effect on survival; in diabetics, the risk for death from coronary artery disease is 2- to 4-fold higher [53]. Demographic statistics underline the role of diabetes in exacerbating coronary disease. In Germany, a diabetic suffers a heart attack every 19 minutes. Additionally, the risk of myocardial infarction (MI) for diabetics diagnosed within 10 years without a preceding MI is equal to the risk level of nondiabetics with a previous MI [53]. 24-hour mortality is also considerably higher following MI surgery in diabetic patients (14%) as compared to nondiabetic patients (5%), according to the “Münchner” MI database [54, 55]. Also, the numbers of end-stage renal disease are dramatically higher in patients with diabetes, also with a clear correlation with age [56].

The upward trend in diabetic prevalence is exemplified in the recent update to the GBD, which shows that diabetes and related metabolic diseases have increased in prevalence and incidence between 1990 and 2015 [17]. Diabetes, defined as high fasting blood glucose, moved from rank 7 to 3 in the list of global health risk factors. Diabetes-associated risk factors also moved up in rank, hyperlipidemia from rank 9 to 6, and obesity from rank 7 to 4 [17]. The increasing prevalence of these risk factors demonstrates the impact of modern lifestyle changes (e.g., unhealthy diet, physical inactivity, and probably environmental triggers) in both Western and industrializing societies. Hyperlipidemia and diabetes are both classical cardiovascular risk factors that are connected through an underlying association with endothelial dysfunction [57, 58]. In addition, they are often both present alongside arterial hypertension, a strong trigger for endothelial dysfunction [59]. A meta-analysis revealed a hazard ratio for cardiovascular mortality for screen-detected diabetes of 3.42 (95% confidence interval (CI): 2.23-5.23) [60]. In a population-based study (*n* = 631), T2DM was cross-sectionally associated with both endothelial dysfunction and low-grade inflammation explaining approximately 43% of the increase in cardiovascular mortality risk in the diabetic group [61]. Markers of inflammation (CRP, IL-6, and TNF-*α*), white blood cell count (as a general parameter of the immune system state), and body mass index (surrogate for the risk factor obesity) show a positive correlation with increasing HbA1c values (Figure 1(e)) [62]. Vice versa, increased markers of inflammation are associated with higher risk for type 2 diabetes development [63]. Hazard ratios for serum CRP levels as well as IL-6 at baseline and risk of T2DM in male and female population were 1.26 (95% confidence interval (CI): 1.16-1.37) and 1.31 (95% confidence interval (CI): 1.17-1.46), respectively (22 cross-sectional studies, *n* = 40,735 subjects for CRP; 10 cross-sectional studies, *n* = 19,709 subjects for IL-6) [63]. Of note, in diabetic and nondiabetic patients with symptomatic chronic heart failure, the HbA1c level is an independent progressive risk factor for cardiovascular death, hospitalization for heart failure, and total mortality [64]. The hazard ratios per 1% higher HbA1c levels (adjusted for age and sex) of the primary composite outcome (CV death or HF hospitalization), cardiovascular (CV) death, hospitalization for worsening heart failure (HF), or death from all causes were 1.25 (95% confidence interval (CI): 1.20-1.31), 1.24 (95% confidence interval (CI): 1.17-1.31), 1.25 (95% confidence interval (CI): 1.19-1.31), and 1.22 (95% confidence interval (C): 1.16-1.29), respectively (*n* = 2,412) [64]. Cardiovascular complications in diabetes mellitus are associated with oxidative stress [65, 66], as are hypercholesterolemia and arteriosclerosis [67, 68].

## 2. The Molecular Triggers of Cardiovascular Disease

### 2.1. Glucotoxicity, AGE/RAGE Signaling, RAAS Activation, and Vasoconstrictors

The mechanism by which diabetes causes cardiovascular complications is complex, but damage to the vascular endothelium is a clear contributor [69]. Proteins in the plasma and cell membrane are altered by chronic exposure to hyperglycemia through the process of nonenzymatic glycosylation, leading to the attachment of glucose molecules. Advanced glycation end-products (AGEs) are formed [70] which then proceed to inactivate nitric oxide (^·^NO), leading to impairment of endothelium-dependent vasodilation [71]. Studies in diabetic rats have found increased AGE/RAGE signaling [72], impairment of ^·^NO/cGMP signaling [73], and also an association with NADPH oxidase-induced oxidative stress and vascular complications [74]. Some of the vascular dysfunction is attributable to a vital crosstalk between oxidative stress and AGE/RAGE components [74–76] leading to increased inflammation [77–79]. In accordance with this concept, the normalization of mitochondrial ROS formation prevented hyperglycemic damage by decreasing AGE/RAGE signaling [76]. Further support comes from observations that macrophages from gp91phox-deficient mice showed reduced responses to AGE stimulation providing a direct link between AGE/RAGE signaling and NADPH oxidase expression/activity [74]. In the same study, a connection between AGE/RAGE signaling and inflammation via increased VCAM-1 and tissue factor expression was demonstrated upon treatment of human endothelial cells with AGE. Furthermore, in the STZ model of diabetes in rats, increased expression of TNF-*α*, IFN-*γ*, and ROS-producing enzymes was normalized upon multiple antioxidant therapy [80], further strengthening the connection between diabetes-induced vascular damage and inflammation. However, the disturbed vascular responses to short-term hyperglycemia are comparable to those seen in the long term, suggesting that AGE/RAGE signaling is not the only mechanism [81]. Aside from AGE and diacylglycerol (DAG) formation, increased oxidative stress has been observed in models of diabetes. In particular, superoxide (O_2_^·–^) is known to contribute to the inhibition of eNOS through depletion of a critical cofactor, leading to uncoupling (reviewed in [82]). Several other pathways are also thought to contribute to the impaired vascular function and thereby increased cardiovascular risk associated with diabetes: activation of protein kinase C, increased metabolization of glucose via hexosamine metabolism leading to increased transcription of genes of inflammatory cytokines and PAI-1, and increased metabolization of glucose to sorbitol by the polyol pathway [83].

Activation of the renin-angiotensin-aldosterone system (RAAS) is also present in the setting of diabetes, as evidenced by higher production of DAG as well as vasoconstrictive compounds such as endothelin-1 and angiotensin-II [84]. Importantly, the activation of RAAS is also closely related to endothelial dysfunction [85, 86] and DAG is a potent activator of protein kinase C [87, 88], which leads to subsequent activation of NADPH oxidase [89]. This RAAS-induced production of superoxide was demonstrated in both clinical [90] and translational studies [91] and is further supported by evidence that suppression of RAAS prevents the progression of T2DM in hypertensive patients [92]. Thrombocytes from diabetics are larger in size, express more glycoprotein receptors on the surface, and are more prone to aggregation in response to a variety of stimuli (for review, see [93]). Another factor for consideration in the vascular consequences of diabetes is the impaired fibrinolytic activity of the endothelium in this setting [94].

### 2.2. Oxidative Stress as a Molecular Trigger of Cardiovascular Disease

Ohara et al. and Harrison and Ohara were the first to describe the role of oxidative stress in the development and progression of cardiovascular disease using an experimental model of hypercholesterolemia [95, 96]. In the intervening years, there has been recognition that the majority of cardiovascular diseases can be characterized as having an imbalance between the formation of ROS and ROS-degrading antioxidant systems [67, 97]. This imbalance leads to the accumulation of superoxide, hydrogen peroxide, and other products such as peroxynitrite and hypochlorous acid and a deviation from the steady state [98]. We have previously reviewed the molecular mechanisms of endothelial (vascular) dysfunction in detail, including the involved sources of ROS and their targets, the “redox switches” in the ^·^NO/cGMP pathway, and related signaling cascades [77, 99, 100]. There are also activation pathways between different ROS sources in a crosstalk fashion [101–103], which was mainly demonstrated for the NOX-2/mitochondrial axis [104, 105], eNOS uncoupling, and xanthine dehydrogenase/oxidase conversion [77, 78], where “ROS-induced ROS formation” can be found as originally reported for self-triggered mitochondrial ROS amplification [106]. More recently, this redox crosstalk concept was extended; in AT-II-induced hypertension, NOX-2 activation triggers Sirt3 S-glutathionylation leading to acetylation of vascular SOD2 and reduced SOD2 activity, all of which resulted in elevated mitochondrial superoxide, diminished endothelial nitric oxide bioavailability, and aggravation of hypertension [107, 108]. This concept was even shown to be operative in tobacco smoke-induced vascular oxidative stress, endothelial dysfunction, and hypertension [109]. In short, there is an indispensable role played by oxidative stress in the development of cardiovascular disease, both mediated somewhat by the uncoupling of endothelial nitric oxide synthase (eNOS) [77], making eNOS uncoupling a hallmark of most cardiovascular diseases [110, 111]. The functional manifestation of eNOS uncoupling is endothelial dysfunction, measurable via acetylcholine-dependent or flow-mediated dilation (FMD), making endothelial dysfunction an important, measurable, and early predictor for cardiovascular events [68, 112].

Because of the near-ubiquitous presence of oxidative stress in cardiovascular disease, an important link has been forged between oxidative stress and cardiovascular prognosis, supported by data from large clinical trials. A study of 636 individuals showed a positive correlation between levels of glutathione peroxidase-1, an antioxidant enzyme, and cardiovascular event-free survival [113]. Another study found that oxidative serum stress markers D-ROM (derivatives of reactive oxygen metabolites, indicative of ROS levels) and total thiol levels (representative of the total redox state) were strongly and independently associated with all-cause and CVD mortality in 10,622 men [114]. Lastly, a meta-analysis of 14 clinical trials spanning more than 1,900 participants yielded evidence that levels of 8-hydroxy-2-deoxyguanosine (a marker of oxidative DNA damage) were increased in patients with cardiovascular disease [115]. Additionally, there have been a number of small cohort clinical studies. These include evaluation of the differential effects of vitamin C on the flow-mediated dilation (FMD) in patients with high or low ROS burden [68]. Other studies utilizing FMD demonstrate significantly impaired response and lower levels of reduced circulating glutathione in smokers (*n* = 52) versus controls [116], a positive correlation between FMD and superoxide dismutase activity (a surrogate of antioxidant defense state), and negative correlations between FMD and oxidized low-density lipoprotein (oxLDL, oxidative stress marker and initiator of atherosclerosis) as well as asymmetric dimethylarginine (ADMA, inhibitor of eNOS and marker for cardiovascular risk) levels in 59 patients with chronic kidney disease (Figure 1(f)) [117]. In sleep apnoea, negative correlations between reactive hyperemia index (a measurement of vascular function) and malondialdehyde/8-oxo-deoxyguanosine levels were found in a study of 69 patients versus controls [118].

Large clinical trials for nonselective antioxidant therapy (mainly vitamin C and E, chronic high dose oral administration) have been conducted and have somewhat surprisingly failed to show any health benefit for the treatment of cardiovascular disease [119, 120], with only few exceptions such as the European Prospective Investigation into Cancer- (EPIC-) Norfolk study with measurement of vitamin C plasma levels of all participants [121] and small-cohort studies using acute administration (mainly infusion) of vitamins (reviewed in [8, 9]). Also, the synthetic antioxidant NXY-059 failed to prove benefits in stroke patients upon costly development and clinical studies [122]. Therefore, the focus has shifted to site- and source-specific antioxidant therapy (e.g., Nrf2 agonists, myeloperoxidase inhibitors, and monoamine oxidase inhibitors) through which we may glean better understanding of these antioxidant systems [120, 123–125].

The free radical theory of aging was first expressed by Harman as early as 1954 [126]. It was founded on the basis that as aging was a universal phenomenon, so must be the contributing factors that drive the process. At first, the focus of his attention was on the hydroxyl radical and molecular oxygen [127]. The theory was then extended to include mitochondria, which are now known to be the most abundant cellular source of ROS with good evidence that they are a major source of ROS in aging tissues [128, 129]. Mitochondrial DNA damage accumulates in the aging cell leading to mitochondrial dysfunction [130] and aging-related cardiovascular and neurodegenerative diseases [35, 131]. The current iteration of the free radical theory of aging is superoxide which accumulates in an age-dependent manner and rapidly consumes ^·^NO, leading to a reduction in the availability in the endothelium and subsequent impaired vasorelaxation [47, 132]. Deletion and/or overexpression of a single antioxidant enzyme (SOD2^+/-^ or SOD2^tg^, GPx-1^−/−^, GPx-4^−/−^ or MsrA^−/−^, SOD1^tg^, and catalase^tg^) have not yielded a definitive association with shortened or lengthened lifespan [133], with the exception of mice completely deficient in SOD2, which typically do not live to term [134, 135]. Another exception lies in the overexpression of Trx1, which increased lifespan and stress resistance [136]. Double gene deletion combinations of SOD1 alongside another antioxidant gene resulted in reduced life expectancy of mice [133, 137]. In summary, there is substantial evidence for a contribution of oxidative stress to shortening of healthspan [138–141] and to detrimental effects on physiological organ function, vascular phenotype, and inflammation [142, 143].

Endothelial cells are activated in response to hyperglycemia, leading to increased expression of endothelial proteins and generation of proinflammatory metabolites. This proinflammatory phenotype is conducive to adhesion of leukocytes and monocytes to the endothelium. Activation of endothelial cells in diabetes may result in a decreased NO bioavailability, which precedes disturbance in endothelial function and the decrease in coronary flow reserve. Cellular activation of the diabetic endothelium may be a key event conferring the characteristic decreased plaque stability and rupture associated with thrombus formation and vessel occlusion [144]. High plasma glucose levels increase the formation of electron-transferring compounds such as NADPH leading to increased formation of ROS. This in turn will lead to oxidative protein modifications such as 3-nitrotyrosine formation [145], oxidative disruption of the zinc-sulphur cluster and uncoupling of eNOS [146] as well as increased levels of toxic aldehydes [147], all common features of diabetes. Additionally, the increased NADPH oxidase-derived ROS leads to oxidative depletion of tetrahydrobiopterin (BH_4_), a critical prerequisite for eNOS uncoupling. Importantly, eNOS uncoupling is a mediator of endothelial dysfunction, which in turn is a hallmark in the early stage of diabetes mellitus [148]. However, through overexpression of the BH_4_ synthesizing enzyme GTP-cyclohydrolase, BH_4_ levels can be rescued and eNOS uncoupling prevented in diabetic mice [149]. In diabetic patients, acute infusion of BH_4_ recoupled eNOS and improved endothelial function [148]. Supplementation with lipoic acid or vitamin C also improved endothelial function in diabetic subjects [150]. Complementary to this, antioxidants have been shown to have a beneficial effect on endothelial function [147, 150–152].

### 2.3. Inflammation as a Molecular Trigger of Cardiovascular Disease

Inflammation is an important player in both chronic disease and cardiovascular disease. Chronic kidney disease, nonalcoholic fatty liver disease, and neurodegeneration are all either triggered by or associated with low-grade inflammation [8, 9, 101]. Low-grade inflammation is also a contributor to the hallmark oxidative stress associated with most cardiovascular disease [7, 100, 153], representing a cardiovascular risk factor [19] that can be targeted by pharmacological treatment [154]. Systemic lupus erythematosus, rheumatoid arthritis, and psoriasis are autoimmune diseases that bridge the gap between chronic and cardiovascular disease, as they possess increased cardiovascular risk [155–158]. Furthermore, psoriasis has even been characterized as an independent risk factor for cardiovascular disease [4] where patients presenting with psoriasis or associated inflammatory arthritis are recommended by the European League Against Rheumatism to undergo cardiovascular therapy [159]. In line with this, systolic blood pressure of patients with rheumatoid arthritis or psoriasis was reduced by immune-suppressive therapy [160]. The basis in this recommendation lies in the presentation of endothelial dysfunction found in psoriatic patients as well as those with rheumatoid arthritis [161, 162]. Since recent research has revealed links between inflammatory signaling networks and the brain, autonomic nervous system, and spleen in both atherosclerosis and myocardial infarction [163], it is not surprising that additional studies found that targeted anti-inflammatory therapy lowered cardiovascular mortality in psoriasis (IL-17/IL-23 axis) [164–166], systemic lupus erythematosus (IL-17A signaling) [167], and rheumatoid arthritis (IL-6, TNF-*α*, and IL-17A cascades) [168, 169]. Several of the aforementioned cytokines (e.g., interleukins IL-6, IL-1*β*, IL-17A, and tumor necrosis factor alpha (TNF-*α*)) are positively correlated with cardiovascular events [7]. Accordingly, clinical trials of IL-1 receptor blockers or antagonists (anakinra, rilonacept, canakinumab (CANTOS trial), and gevokizumab) in patients with cardiovascular disease are underway or already completed [170]. Also, recent discoveries of Genome-Wide Association Study (GWAS) on risk loci in a large population cohort (*n* = 194,427 participants) suggest an important role of arterial wall-specific inflammation processes in the development and progression of coronary artery disease; for example, SNPs in or near genes involved in cellular adhesion, leukocyte migration and atherosclerosis (PECAM1, rs1867624), and coagulation and inflammation (PROCR, rs867186 (p.Ser219Gly)) were identified [171]. Also, other recent GWAS data contained several risk loci that are linked to cardiovascular inflammation [172]. Endothelial microparticles (EMPs) are vesicles with a size range of 0.1-1.0 *μ*m that are released upon endothelial cell activation or apoptosis and promote atherosclerosis by the induction of oxidative stress and vascular inflammation [173]. Therefore, the pleiotropic anti-inflammatory effects of the most successful cardiovascular drugs (e.g., statins, ACE inhibitors, and AT1R blockers) as well as new antidiabetic drugs (gliptins, GLP-1 analogues, and SGLT2 inhibitors), which have been found to possess potent immune-suppressant properties in experimental analyses and postmarket launch, represent valuable therapeutic add-ons [8, 174, 175]. All these data support a link between cardiovascular disease and chronic autoimmune diseases through inflammation (also reviewed in [155, 157]). It is worth noting that also negative data exists as shown by the recent study on low-dose methotrexate for the prevention of atherosclerotic events (*n* = 4,786 patients) [176]. The study was stopped earlier since methotrexate did neither reduce levels of IL-1*β*, IL-6, and CRP nor the number of cardiovascular events and most importantly caused higher incidence of non-basal-cell skin cancers as compared to placebo.

The presence of low-grade inflammation is also notable as immunosenescence within the aging process which contributes to a higher prevalence of metabolic and cardiovascular complications in the elderly [177]. The clear overlap between the risk factors of cardiovascular diseases and diabetes, particularly regarding inflammation, implies that the immunosenescence could represent a key player for aging-associated diseases and increased comorbidity in the elderly [22–24]. Therefore, normalizing the chronic inflammatory phenotype and additionally, targeting mitochondrial oxidative stress (mtROS) could represent a promising strategy to increase healthspan [178]. mtROS formation increases with advancing age [45] and leads to the activation of immune cells and their phagocytic NADPH oxidase, thereby stimulating cytokine release and the inflammasome [78, 179–183]. The hypothesis that targeting mitochondrial ROS could be a strategy for suppression of the chronic inflammatory phenotype of advancing age was recently supported by a study in an animal model for metformin-dependent AMP-activated protein kinase activation. Elderly animals had an increased lifespan and healthspan as a result of suppression of inflammation and oxidative damage [184]. As highlighted in the preceding sections, there is also a vital crosstalk between oxidative stress and AGE/RAGE components [74–76] leading to increased inflammation [77–79]. The crosstalk between oxidative stress and inflammation will be discussed in more detail in the subsequent section.

Also, hypertension, a leading cardiovascular risk factor for global mortality and disease burden, is now seen as an inflammatory disease [185]. The molecular mechanisms are explained in detail by experimental animal data in the following section. Of note, more recently autoimmune mechanisms were reported as the driving force of hypertension leading to infiltration of immune cells, oxidative stress, and stimulation of the intrarenal angiotensin system by the activation of the innate and adaptive immunity [186, 187]. This new concept is mainly based on the discovery of autoantigens (e.g., isoketal-modified proteins by animal and human studies).

### 2.4. Interplay of Oxidative Stress and Inflammation

Inflammatory processes clearly connect vascular dysfunction and cardiovascular diseases such as arterial hypertension, hypercholesterolemia, and coronary artery disease [7, 153]. Additionally, recent data provide a basis for this tight association between redox regulatory pathways and inflammation via redox activation of immune cells by mitochondrial superoxide/hydrogen peroxide and the subsequent activation of the phagocytic NADPH oxidase (NOX-2) [78, 188]. Cultured T cells isolated from spleens of in vivo angiotensin-II- (AT-II-) treated mice showed increased mitochondrial superoxide/hydrogen peroxide formation and thereby increased TNF-*α* production, as well as heightened energy metabolism via increased ATP production. All of these alterations were corrected by ex vivo treatment with mitoTEMPO, a mitochondria-targeted antioxidant and SOD mimetic [183]. Mitochondrial superoxide/hydrogen peroxide formation is a strong trigger (via the redox crosstalk [77, 101]) for NOX-2 activation, which is in turn critical for the activation, recruitment, and infiltration of myelomonocytic cells [189, 190] and T cells [191]. Evidence for this was demonstrated by Wenzel et al. by adoptive cell transfer of monocytes in mice with deficiency of myelomonocytic cells. Myelomonocyte-deficient mice were resistant to the effects of AT-II infusion, but lost this resistance upon transfer of monocytes from wild-type mice, but not upon transfer of monocytes from gp91^phox-/-^ (Nox2^−/−^) [189]. Similarly, Guzik et al. have shown that adoptive cell transfer of T cells (but not B cells) from wild-type mice but not from p47^phox-/-^ mice restored hypertension and all adverse effects of AT-II infusion in RAG1^−/−^ mice with deficiency in most immune cells [191]. A substantial role of B cells for the development of hypertension by AT-II treatment was also shown [192]. Conversely, the normalization of blood pressure in hypertensive humanized mice suppressed infiltration of immune cells [193], compounding evidence that inflammation is a key player in redox-regulated vascular dysfunction via redox crosstalk of mitochondria and NOX-2 in immune cells and through various other signaling events like fatty acid metabolism (e.g., formation of leukotrienes or oxLDL). More recently, a redox-dependent crosstalk between inflammatory and coagulation pathways was described for experimental hypertension that contributes to increased tissue factor levels, leukocyte-platelet interactions, vascular infiltration of immune cells, oxidative stress, endothelial dysfunction, and blood pressure, all of which were normalized by pharmacological, genetic, or immunological blockade of VCAM-1, tissue factor, thrombin, FXI, GPIb*α*, and Mac-1 signaling [194]. Consideration of platelet functional state for estimation of vascular inflammation and cardiovascular risk may become even more attractive since the mean platelet volume shows associations with hypertension and rheumatoid arthritis [195] and clinical impact as a risk marker, although technical problems still prevent the broad clinical use of this parameter [196]. Details on the interaction of immune cells (mainly monocytes/macrophages), platelets, and vascular cells with consideration of redox-based mechanisms can be found elsewhere [100, 197–199].

**Figure 2:**
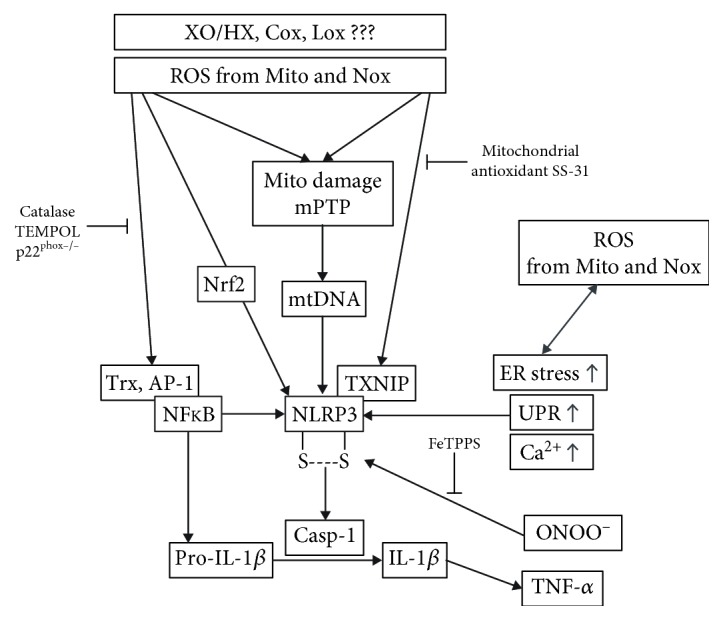
Crosstalk between oxidative stress and inflammation. Interaction between ROS and mediators of inflammation exemplified by redox regulation of the transcription factors Nrf2 and NF-*κ*B and the NLRP3 inflammasome as well as their downstream targets. In several pathways, inhibitory effects by antioxidants or genetic deletion of ROS sources are indicated. Modified from Wenzel et al. [100] (with permission from Elsevier. © 2017 Elsevier Inc.; all rights reserved).

Also, protein oxidation, oxidative DNA damage and oxidized nucleotides, redox activation of protein kinases (e.g., ERK1/2, JNK), and redox inhibition of protein phosphatases regulate inflammatory processes. For example, mtROS can increase mitochondrial permeability (by opening of the mitochondrial permeability transition pore) with subsequent release of (oxidized) mtDNA, which represents a damage-associated molecular pattern (DAMP) and initiates a process called “sterile inflammation” (Figure 2) [100, 200]. Other examples for the contribution of ROS to inflammation pathways are redox-dependent formation of protein complexes (e.g., p53-JNK, Nrf2-Keap1), redox regulation of mediators of inflammation (e.g., HMGB1, S100 proteins, and DAMPs), and redox modulation of transcription factors involved in inflammatory pathways (e.g., Nrf2, AP-1, NF-*κ*B, and HIF-1*α*) [201–203]. Additional recent evidence linking oxidative stress, inflammation, and the development of hypertension lies in the formation of isoketals, highly reactive compounds that form protein adducts that can then trigger immune cell infiltration into the vasculature and high blood pressure. [204]. The cellular redox potential controls the migration of monocytes/recruitment of macrophages [205]. Finally, redox modifications of MAPK phosphatase 1 [206] and Slingshot-1L-binding protein 14-3-3zeta [207] as well as changes in the S-glutathionylation pattern [208] represent other potential redox regulatory mechanisms. This crosstalk between oxidative stress and inflammation in general is supported by evidence for specific redox regulation of the cytokine release controlling NLRP3 inflammasome (Figure 2) [179–181, 209–211], the central organizer of inflammation high-mobility group box 1 (HMGB1) [203, 212–215], and the antibacterial process of neutrophil extracellular trap (NET) formation [216–221] as well as transcription factors and other essential mediators of inflammation (reviewed in [100]). In particular, the NLRP3 inflammasome and HMGB1 represent major “redox hubs” of the inflammation cascade and are regulated by redox-sensitive cysteine residues. The crystal structure of NLRP3 contains a highly conserved disulfide bond connecting the PYD domain and the nucleotide-binding site domain, which is highly sensitive to altered redox states [211]. Reduction of all thiols in HMGB1 makes it a chemokine, a disulfide bond between Cys23 and Cys45 converts it to a proinflammatory cytokine, and oxidation of all thiols makes it inactive [215].

Direct molecular proof for the interplay of oxidative stress and inflammation comes from studies of mice with genetic modulation of antioxidant defense or ROS-producing enzymes, all of which show an alteration of the inflammatory phenotype (Table 1). Our own observations of a correlation between a marker of inflammation (CD68 staining) and overall vascular ROS formation (dihydroethidine (DHE) staining) revealed an age-dependent progressive phenotype of low-grade inflammation in glutathione peroxidase-1- (GPx-1-) deficient mice [46, 143]. Since global vascular ROS formation was correlated with mitochondrial ROS formation and all other parameters were linked to global vascular ROS formation, one can assume that mitochondrial and cellular ROS formation significantly contributes to eNOS dysregulation/uncoupling, vascular function, and low-grade inflammation during the aging process [46, 143]. White blood cells from mice with heterozygous mitochondrial superoxide dismutase (MnSOD or SOD2) deficiency showed higher NOX-2 activity and endothelial dysfunction, all of which were further aggravated upon challenges with AT-II [78]. Likewise, genetic deletion of heme oxygenase-1 (HO-1) caused induction of NOX-2 at the protein level, increased levels of vascular ROS, and endothelial dysfunction, all of which were further aggravated by AT-II-induced hypertension [222]. HO-1 deficiency also caused increased expression of the C-C chemokine receptor type 2 (CCR2 or CD192), leukocyte rolling, and adhesion to the endothelial cell layer and numbers of infiltrated neutrophils and monocytes. More examples for pro- or anti-inflammatory effects by genetic modulation of antioxidant defense or ROS-producing enzymes can be found in Table 1.

## 3. Anti-Inflammatory and Antioxidant Properties of Dipeptidyl Peptidase-4 (DPP-4) Inhibitors, Glucagon-Like Peptide-1 (GLP-1) Analogs, and Sodium-Glucose Cotransporter 2 (SGLT2) Inhibitors

Current evidence suggests that SGLT-2 inhibitors are more effective than both GLP-1 agonists and DPP-4 inhibitors in reducing the risk of hospitalization for heart failure (HHF) in T2DM (87,162 participants) [223], although the latter showed more potency in reducing HHF in T2DM than sulphonylureas (e.g., glibenclamide) and thiazolidinediones (glitazones) (127,555 patients in the meta-analysis) [224]. According to another large-scale clinical study, incretin-based therapy by GLP-1 agonists or DPP-4 inhibitors showed a trend for decreased risk of hospitalization for heart failure (1,499,650 patients, with 29,741 hospitalized for heart failure) [225]. Both drug classes have cardioprotective effects in models of myocardial infarction [226–229]. In addition, both drug classes provide potent anti-inflammatory properties [230–232], which could be beneficial in many cardiovascular but also neurodegenerative diseases. In the following two subsections, we will present and discuss our recent findings on anti-inflammatory effects of SGLT-2 inhibitors, GLP-1 agonists, and DPP-4 inhibitors in animal models of diabetes type 1/2 as well as experimental sepsis.

### 3.1. DPP-4 Inhibition and GLP-1 Supplementation

Glucagon-like peptide-1 (GLP-1) is a peptide hormone with known anti-inflammatory properties. Endogenous GLP-1 is degraded by the exopeptidase dipeptidyl peptidase-4 (DPP-4) within seconds under physiological conditions. It increases insulin release from beta-cells in the pancreas and consequently its synthetic analogs are used for treatment of type 2 diabetes. Clinical trials, like LEADER (liraglutide) and SUSTAIN-6 (semaglutide), demonstrated that GLP-1 analogs reduce the risk of cardiovascular events in T2DM patients, an effect beyond glycemic control [233, 234]. In the LEADER trial, patients suffering from T2DM were treated with standard therapy or standard therapy plus liraglutide. In this clinical trial, the risk of death from cardiovascular causes nonfatal myocardial infarction or nonfatal stroke was lower with liraglutide than with placebo (hazard ratio, 0.87; 95% confidence interval (CI), 0.78 to 0.97; *p* < 0.001 for noninferiority; *p* = 0.01 for superiority). According to these results, another trial (SUSTAIN-6) found similar results for the GLP-1 analog semaglutide. The primary endpoint (death from cardiovascular causes, nonfatal myocardial infarction, or nonfatal stroke) occurred in 6.6% of the study population in the semaglutide group and in 8.9% in the placebo group (hazard ratio, 0.74; 95% confidence interval (CI), 0.58 to 0.95; *p* < 0.001 for noninferiority) [233, 234]. Other clinical trials investigated the effects of GLP-1 on cardiovascular disease and demonstrated cardioprotective effects after myocardial infarction [235], reduction of blood pressure [236], and an improvement of endothelial dysfunction [237]. Interestingly, it has been shown that GLP-1 levels in patients with coronary artery disease are reduced [238]. Experimental studies provide first evidence for a modulating effect of GLP-1 on inflammatory cells, and it has been recently published that the GLP-1 degrading enzyme dipeptidylpeptidase-4 (DPP-4) promotes vascular adipose tissue inflammation and insulin resistance in obesity [239], also suggesting a potential role of broader “metabolic alterations” in redox-mediated inflammation. Other studies report vascular protection by DPP-4 inhibition in animal models of atherosclerosis due to ameliorated vascular inflammation [174, 175]. Also, all major complications of experimental nonalcoholic fatty liver disease (NAFLD) and nonalcoholic steatohepatitis (NASH), namely, hepatic and vascular inflammation, were improved by DPP-4 inhibitor therapy by linagliptin and sitagliptin [240]. The therapy with GLP-1 analogs may be also useful for patients with NAFLD or NASH [241]. However, it remains puzzling why clinical trials for DPP-4 inhibitors like saxagliptin failed to improve the cardiovascular outcome or even increased the rate of hospitalization for heart failure [242].

**Figure 3:**
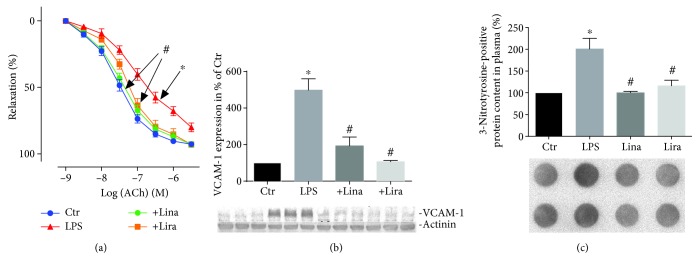
Antioxidant, anti-inflammatory, and vasculoprotective effects by the DPP-4 inhibitor or GLP-1 analog therapy in septic rats. (a) Endothelium-dependent (ACh) relaxation was determined by isometric tension studies in rat aortic ring segments. (b) Aortic expression of inflammatory protein VCAM-1 was assessed by Western blotting analysis and specific antibodies. (c) Levels of 3-NT-positive proteins in plasma were assessed by dot blot analysis and specific antibodies. Representative blots are shown below the densitometric quantification. The data are mean ± SEM from 4-8 (a–c) rats/group. ^∗^*p* < 0.05 vs. the control and ^#^*p* < 0.05 vs. LPS. Adopted from Steven et al. [246] (with permission from Springer-Verlag Berlin Heidelberg. Copyright © 2015, Springer).

Sepsis is an inflammatory disease and can affect the whole organism. Depending on the individual patient's characteristics, a pneumonia can expand to a “systemic inflammatory response syndrome” (SIRS) with a mortality of 40%, making it a leading cause of death in Western countries [243]. In experimental sepsis, the anti-inflammatory effects of GLP-1 analogs and DPP-4 inhibition were recently confirmed [244, 245]. In lipopolysaccharide- (LPS-) induced septic shock, the DPP-4 inhibitor linagliptin improved vascular function and reduced vascular superoxide formation [248]. Linagliptin reduced oxidative burst in whole blood and ameliorated infiltration of white blood cells to the vascular wall. Furthermore, the expression of NADPH oxidase was reduced [245]. In another study, our group demonstrated that DPP-4 inhibition by linagliptin, GLP-1 supplementation by liraglutide, and genetic deletion of DPP-4 improve survival of LPS-induced endotoxemia in mice [246]. In accordance, EPR (electron paramagnetic resonance) spectroscopy studies revealed reduced iNOS activity (nitrosyl-iron hemoglobin (HbNO)) in whole blood of endotoxemic rats under linagliptin and liraglutide therapy [246]. Likewise, mRNA expression of IL-6, iNOS, ICAM-1, MCP-1, TNF-*α*, and VCAM-1 in aortic tissue of endotoxemic rats was suppressed by both drugs, an observation that was accompanied by improved endothelial function and reduced nitrooxidative stress as measured by 3-nitrotyrosine-positive proteins (Figures 3(a)–3(c)) [246]. Importantly, these protective effects are beyond glycemic control since no changes in blood glucose levels were reported.

**Figure 4:**
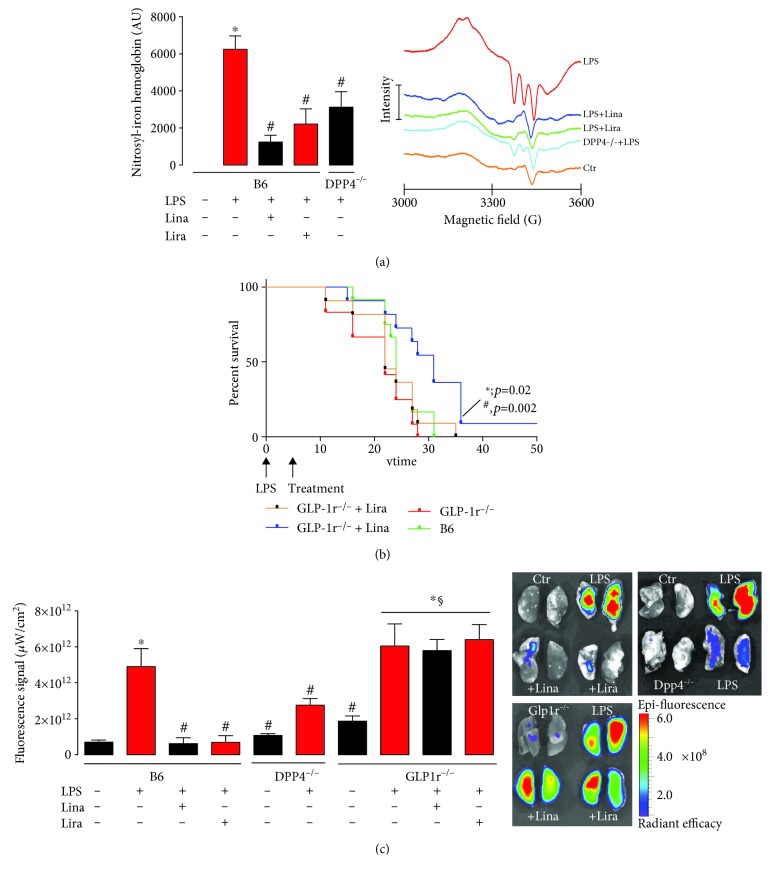
Antioxidant, anti-inflammatory, and vasculoprotective effects by the DPP-4 inhibitor or GLP-1 analog therapy in septic mice. (a) Whole blood Hb-NO levels were determined by electron paramagnetic resonance spectroscopy as a read-out of iNOS activity. (b) Mortality of endotoxemic mice was assessed by Kaplan-Meier curves recording the survival in dependence of time. 17.5 mg/kg LPS or solvent was administrated by i.p. injection. DPP-4 inhibitor (Lina: 5 mg/kg/d s.c. for 3 d) and GLP-1 analogue (Lira: 200 *μ*g/kg/d s.c. for 3 d) treatment was started 6 h after the induction of endotoxemia. (c) Microvascular thrombosis was detected by fluorescence imaging using fluorescent microbeads in endotoxemic wild-type mice, DPP-4^−/−^ mice, and GLP1r^−/−^ mice. Representative images of lungs are shown beside the quantification. The data are mean ± SEM from 6-18 (a), 12 (b), or 4-6 (c) mice/group. ^∗^*p* < 0.05 vs. B6; ^#^*p* < 0.05 vs. B6+LPS; ^§^*p* < 0.05 vs. GLP-1r^−/−^. Adopted from Steven et al. [247] (with permission from John Wiley and Sons. Copyright © 2016, John Wiley and Sons).

Endotoxemic mice suffer from severe thrombocytopenia, which appears in the setting of disseminated intravascular coagulation. Microvascular thrombosis in the pulmonary circulation (detected by fluorescence imaging) led to severe organ damage and increased lactate dehydrogenase (LDH) activity. Vascular function was impaired, and increased markers of inflammation in the aorta and whole blood (leukocyte-dependent whole blood oxidative burst, HbNO) were present (Figure 4(a)) [247]. Treatment with linagliptin (DPP-4 inhibitor) and liraglutide (GLP-1 analogue) as well as genetic deficiency of DPP-4 (DPP-4^−/−^ mice) improved all these parameters. Interestingly, in GLP-1 receptor knockout mice, liraglutide completely and linagliptin partially lost their therapeutic efficiency as demonstrated by survival studies (Figure 4(b)) [247]. Incubation of cultured monocytes with GLP-1 analogues inhibited the monocytic oxidative burst by the elevation of cAMP and increased protein kinase A (PKA) activation as a potential mechanistic explanation of the beneficial GLP-1-dependent signaling effects.

Besides inflammatory cells, inhibition of DPP-4 and GLP-1 might also have impact on thrombocytes. The experiments in endotoxemic animals revealed that the bleeding time was ameliorated by DPP-4 inhibition and GLP-1 supplementation in rats, a first evidence for a role of DPP-4/GLP-1 signaling in hemostasis [246]. Antiaggregatory effects by sitagliptin (DPP-4 inhibitor) on human platelets were shown by Gupta et al., without demonstrating a potential mechanism [248]. Cameron-Vendrig et al. investigated the role of the GLP-1 receptor on platelets in more detail. They reported that the GLP-1 analog exenatide leads to reduced formation of thrombi in mice [249]. The authors demonstrated that the GLP-1 receptor is expressed on platelets and activation of the receptor by GLP-1 analogs increases cAMP levels, which was followed by reduced thrombocyte aggregation in vitro and ex vivo. Sepsis causes disseminated intravasal coagulation (DIC), which is associated with thrombin generation and uncontrolled platelet activation. Under these conditions, clot formation impairs microcirculation and leads to severe end organ damage [250]. The GLP-1 analog liraglutide improved microcirculation in lung tissue by inhibition of overwhelming thrombocyte aggregation in an animal model of septic shock [247]. With our own studies, we could show that liraglutide and linagliptin as well as genetic DPP-4 deficiency prevent DIC and that this protective effect of both drugs is lost in GLP-1 receptor knockout mice (Figure 4(c)) [247]. Increased activity of cAMP/PKA signaling by GLP-1 is a potential mechanism for the antiaggregatory effects of DPP-4 inhibition and GLP-1 analogs.

### 3.2. SGLT2 Inhibition

Therapy with the SGLT2 inhibitor ipragliflozin not only improved hyperglycemia, hyperlipidemia, obesity, hepatic steatosis, and oxidative stress but also various parameters of inflammation in mice with T2DM or T1DM [251, 252]. The SGLT2 inhibitor dapagliflozin ameliorated glucose homeostasis and diabetic nephropathy and decreased markers of inflammation in db/db mice [253]. Various SGLT2 inhibitors (e.g., luseogliflozin, dapagliflozin, and empagliflozin) also beneficially influenced progression of atherosclerosis by anti-inflammatory effects [254–256]. Likewise, SGLT2 inhibitors (e.g., empagliflozin, canagliflozin, ipragliflozin, and luseogliflozin) protected against NAFLD complication by decreasing inflammatory signaling [257–262], and these beneficial effects were even observed in patients with NAFLD or NASH [263, 264].

Empagliflozin is a potent and specific new therapeutic agent in the treatment of type 2 diabetes (T2DM) in the United States [265] and European Union (European Medicines Agency number: EMEA/H/C/002677). It is a member of a class of drugs (SGLT2i) whose mechanism of action is to inhibit the sodium-glucose cotransporter 2 (SGLT2), a protein responsible for the renal reabsorption of >90% of the glucose from the primary urine [266]. SGLT2i are able to prevent hyperglycemic episodes and glucotoxicity by increasing the excretion of glucose [267, 268]. Due to their insulin-independent mechanism of action, SGLT2i are not affected by deteriorating *β*-cell function and desensitization to insulin signaling, as is commonly seen in the elderly [269]. In recent large-scale clinical trials (EMPA-REG), empagliflozin was the only modern antidiabetic drug that was able to reduce overall cardiovascular mortality in diabetic patients at high cardiovascular risk [270, 271].

**Figure 5:**
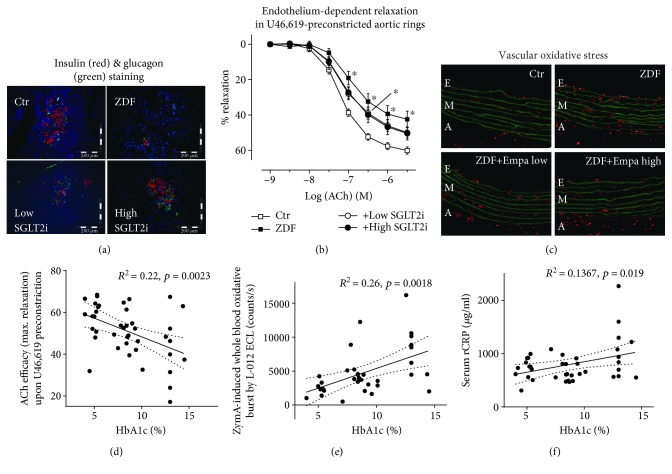
Antioxidant, anti-inflammatory, and vasculoprotective effects by empagliflozin therapy in ZDF rats. (a) Representative (immuno)histochemical stainings of pancreatic tissue for insulin, glucagon, and nuclei using fluorescent antibodies and dyes. (b) Improvement of endothelium-dependent relaxation by the vasodilator acetylcholine (ACh) in U46,619-preconstricted aortic ring segments. (c) Dihydroethidium (DHE, 1 *μ*M) fluorescence microtopography was used to assess the effects of SGLT2i treatment on whole vascular wall ROS production, and representative microscope images are shown (red fluorescence indicates ROS formation whereas green fluorescence represents basal laminae autofluorescence). Linear regression analysis for correlations between HbA1c and endothelial function (ACh efficacy, d), zymosan A-induced whole blood oxidative burst (e), and serum CRP levels (f) using a total of 35-41 rats. ^∗^*p* < 0.05 vs. the control. Adopted from Steven et al. [272] (with permission from Elsevier. © 2017 The Authors. Published by Elsevier B.V.).

Empagliflozin is very effective in preventing glucotoxicity, including the increased methylglyoxal levels, formation of AGE, and induction of RAGE-dependent signaling that are typically seen in diabetic models. In the Zucker Diabetic Fatty (ZDF) rat model of diabetes, empagliflozin efficiently restored glycemic control. Immunohistochemical and histochemical analyses suggest that this restoration was due to the preservation of beta-cells [272]. This preservation also extended to the *α*-cells of the pancreatic islets and their glucagon content, suggesting that empagliflozin may affect glucose metabolism in a dual-pronged manner (Figure 5). Importantly, empagliflozin also improved the function of thoracic aortic segments, which coincides with linear regression analysis that revealed a significant inverse correlation between endothelial function and HbA1c. Empagliflozin treatment was also seen to have reduced oxidative stress in the aorta and blood of diabetic rats, as evident in correlations between leukocyte-dependent oxidative burst and HbA1c (Figure 5). Other's previous data showed that AGE/RAGE signaling triggers low-grade inflammation [79]. It is then possible that dynamic suppression of AGE formation and RAGE signaling, obstructing ROS-induced inflammatory pathways, and decreased epigenetic activation of inflammatory genes may all contribute to empagliflozin's anti-inflammatory properties. This is supported by our own data showing that inflammation and glucotoxicity (AGE/RAGE signaling) were epigenetically prevented by SGLT2i treatment as demonstrated by ChIP analysis. We found that there was decreased activation of *NOS2* and *IFNγ* (for *RAGE* by trend) due to decreased H3K4me3 in the promoter regions of the genes in kidneys of SGLT2i-treated ZDF rats, but it is not currently possible to discern whether this is a consequence of improved glucose regulation or a specific property of SGLT2i. However, additional evidence for pleiotropic activity by SGLT2i comes from improved eNOS function in hyperglycemic endothelial cells undergoing empagliflozin therapy, even independent of glucose lowering. This effect was also shared by DPP-4 inhibitor sitagliptin and partially by the RAGE antagonist FPS-ZM1 [272].

We reported similar antidiabetic, antioxidant, anti-inflammatory, and vasculoprotective effects of empagliflozin treatment in rats with streptozotocin- (STZ-) induced T1DM [267]. Treatment with empagliflozin importantly reversed the proinflammatory phenotype of diabetes as well as the glucotoxicity (AGE/RAGE). In diabetic rats, empagliflozin expectedly reduced blood glucose levels but it also normalized endothelial function in aortic rings and reduced oxidative stress in aortic vessels (dihydroethidium staining) and in blood (phorbol ester/zymosan A-stimulated chemiluminescence).

## 4. Conclusion

**Figure 6:**
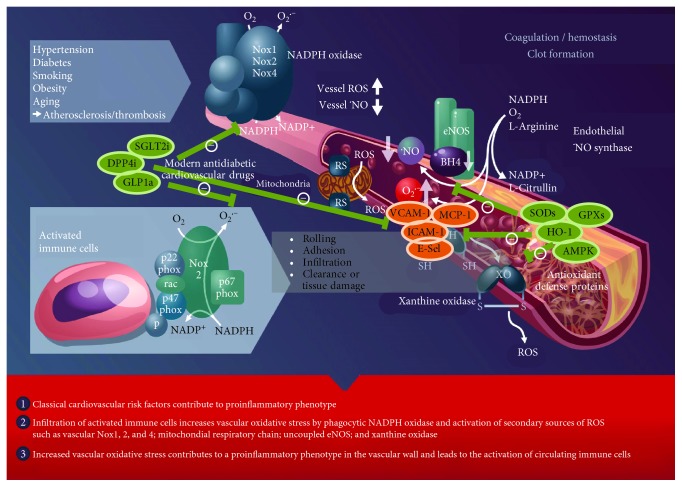
Oxidative stress and inflammation trigger atherothrombosis. The scheme illustrates the activation of immune cells and recruitment to vascular tissues by classical cardiovascular risk factors, leading to the activation of secondary vascular ROS sources such as NADPH oxidase (Nox1, Nox2, and Nox4), xanthine oxidase (conversion of the dehydrogenase (XDH) to the oxidase (XO) form), mitochondria (via mitochondrial redox switches (RS)), and uncoupled eNOS (oxidative depletion of tetrahydrobiopterin (BH4) and other redox switches), all of which contribute to vascular dysfunction, progression of atherosclerosis, and thrombus formation. Adhesion molecules and chemokines (VCAM-1, ICAM-1, E-selectin, and MCP-1) play an important role for leukocyte recruitment to vascular tissues leading to the secondary damage. Endogenous antioxidant defense proteins (e.g., superoxide dismutases, glutathione peroxidases, heme oxygenase-1, and AMP-activated protein kinase) interfere with oxidative damage and redox-dependent inflammatory processes. Also, modern antidiabetic cardiovascular drugs (e.g., SGLT2 inhibitors, DPP-4 inhibitors, and GLP-1 analogs) suppress inflammatory and adverse redox pathways and thereby decrease cardiovascular risk. Modified from Steven et al. [8]. Open access article distributed under the Creative Commons Attribution License (CC BY 4.0).

Cardiovascular disease is the leading cause of mortality in industrialized nations. Smoking, unhealthy nutrition, aging population, lack of physical activity, arterial hypertension, or diabetes promote cardiovascular disease like myocardial infarction or stroke (Figure 6). It is multifactorial and encompasses a multitude of mechanisms, such as eNOS uncoupling, reactive oxygen species formation, abnormal calcium homeostasis, signaling associated with a deleterious phosphorylation pattern, and futile counterregulatory mechanisms at the humoral, cellular, and tissue levels. Previous data also implicate immune cells and inflammatory signaling as essential contributors to cardiovascular disease (for review, see [7]). Protective effects are discussed for important endogenous antioxidant enzymes as well as for modern drugs with pleiotropic antioxidant and anti-inflammatory properties. Three highly promising classes of modern antidiabetic cardiovascular drugs are highlighted (SGLT2 inhibitors, DPP-4 inhibitors, and GLP-1 analogs). The present review defines the current knowledge connecting the immune system with cardiovascular disease, allowing not only for more integrative therapies using multidisciplinary expertise but also potential treatments for the associated comorbidities. In the future, the modulation of epigenetic pathways may represent an important future strategy due to the observed global changes in H3K4 trimethylation which are associated with high body weight and T2DM [273]. Additionally, increased promoter H3K9me2 in genes associated with autoimmune and inflammation-related pathways (transforming growth factor-beta (TGF*β*), nuclear factor-*κ*B, p38 mitogen-activated protein kinase, toll-like receptor, and interleukin-6) are present in diabetic patients implying anti-inflammatory potential of epigenetic modulators [274]. An additional field open for investigation lies in the comparison of drugs with similar primary pharmacological effects (e.g., blood pressure lowering), with and without known pleiotropic effects (e.g., anti-inflammatory or antioxidant components) in order to discern the role of this pleiotropy. This data would be valuable in treating patients, especially in conjunction with prescreening for markers of inflammation and/or oxidative stress—paving the way toward personalized medicine in cardiovascular disease therapy.

## Figures and Tables

**Table 1:** Molecular proof for a crosstalk between oxidative stress and inflammation in mice or cell culture with genetic modulation of antioxidant defense or ROS-producing enzymes.

Antioxidant or prooxidant protein	Phenotypic changes	Reference
Glutathione peroxidase-1 (GPx-1), homozygous deficiency	(1) Increased marker of inflammation (CD68 and F4/80 staining) and increased vascular and mitochondrial ROS formation (dihydroethidine (DHE) staining) (2) Increased cardiac and vascular NOX-2 activity (3) Vascular dysfunction and eNOS uncoupling (4) Age-dependent progressive phenotype of low-grade inflammation, impaired redox balance, and vascular dysfunction (5) Increased adhesion of leukocytes to cultured GPx-1-deficient endothelial cells	[46, 143]

GPx-1 deficiency in human microvascular endothelial cells (HMVEC)	(1) Intercellular adhesion molecule-1 (ICAM-1) and vascular cell adhesion molecule-1 (VCAM-1) expression via ROS and NF-*κ*B (2) More TNF-alpha-induced ROS production and activation of ERK1/2 and JNK (that was suppressed by GPx-1 overexpression)	[275]

GPx-1/2 deficiency, homozygous double knockout	(1) Loss of control of inflammatory response in the intestinal mucosa (2) Symptoms and pathology consistent with inflammatory bowel disease (colitis) (3) High incidence of mucosal inflammation in the ileum and colon (4) Elevated levels of myeloperoxidase activity and lipid hydroperoxides in the colon mucosa	[276]

Mitochondrial superoxide dismutase (Mn-SOD or SOD2), heterozygous deficiency	(1) Higher NOX-2 activity in isolated white blood cells (2) Endothelial dysfunction (3) Further aggravation of endothelial dysfunction, eNOS uncoupling, and hypertension upon challenges with AT-II	[78]

Cytoplasmic copper/zinc superoxide dismutase (CuZn-SOD and SOD1) homozygous and heterozygous deficiency	(1) More pronounced cognitive impairment and synaptic dysfunction in the Alzheimer disease model (2) Increased amyloid *β* (A*β*) 40/42 oligomers plaque formation in the brain (3) Increased neuronal inflammation by activation of microglia and astrocytes (4) Increased cerebral oxidative damage (measured by 8-oxo-dG)	[277]

Extracellular superoxide dismutase 3 (SOD3), homozygous deficiency and overexpression	(1) Deficiency aggravated ovalbumin-induced allergic airway inflammation in mice as a model of asthma (2) Deficiency increased infiltrated leukocytes and cytokine levels in lung tissue (3) Overexpression decreased inflammatory molecules in lung fibroblasts (chemokine receptor 4 (CCR4), TNF-*α*, TGF*β*, IL-1*α*, and IL-1*β*) (4) Overexpression reduced extracellular matrix molecules (collagen III and syndecan1)	[278]

Heme oxygenase-1 (HO-1), homozygous and heterozygous deficiency	(1) Induction of NOX-2 at the protein level (HO−1^−/−^ > HO−1^+/−^ > WT Ctr) (2) More vascular ROS and endothelial dysfunction (3) Further aggravation upon challenges with AT-II (4) Increased expression of the C-C chemokine receptor type 2 (CCR2 or CD192) (5) More pronounced leukocyte rolling and adhesion to the endothelial cell layer (6) Increased numbers of infiltrated neutrophils and monocytes in aortic tissue (7) Further aggravation of endothelial dysfunction in the setting of diabetes and during the aging process	[222]

Endothelial and myelomonocyte-specific *α*1AMPK deficiency (TekCre and LysMCre mice)	(1) Adverse AT-II effects (endothelial dysfunction and oxidative stress, markers of inflammation and leukocyte rolling/adhesion/infiltration) were further aggravated in LysMCre × *α*1AMPK (2) Adverse AT-II effects (endothelial dysfunction and oxidative stress, markers of inflammation and leukocyte rolling/adhesion/infiltration) were further aggravated in TekCre × *α*1AMPK in a HO-1-dependent manner (3) *α*1AMPK and HO-1 siRNA caused increased expression of VCAM-1 and MCP-1 in cultured endothelial cells, all of which were rescued by treatment with PEG-SOD	[279, 280]

Endothelial cell-specific Nox2 transgenic overexpression	(1) Greater cardiac fibrosis (2) More pronounced left ventricular diastolic dysfunction (3) More inflammatory cells (CD45^+^ and MAC3^+^) and VCAM-1-positive vessels in the myocardium upon challenges with AT-II (4) Increased adhesion of leukocytes to cultured endothelial cells of Nox2 transgenic mice upon challenges with AT-II	[281]

Nox2, homozygous deficiency	(1) Protection against aircraft noise-induced endothelial dysfunction and vascular/cerebral oxidative stress (2) Prevention of aircraft noise-induced increases in plasma IL-6 or cerebral iNOS, CD68, and IL-6 levels	[282]

Nox1, homozygous deficiency	(1) Less diethylnitrosamine- (DEN-) induced liver tumors (2) Decreased TNF-*α* and IL-6 levels upon DEN challenges (3) Protective effects were also seen in macrophage-specific Nox1-deficient mice (but not in those mice with hepatocyte- or hepatic stellate cell-specific Nox1 deficiency or global Nox4^−/−^ mice)	[283]
